# Epigenetic control of Foxp3 in intratumoral T-cells regulates growth of hepatocellular carcinoma

**DOI:** 10.18632/aging.101918

**Published:** 2019-04-21

**Authors:** Qin Liu, Fang Du, Wei Huang, Xiaoyi Ding, Zhongmin Wang, Fuhua Yan, Zhiyuan Wu

**Affiliations:** 1 Department of Interventional Radiology, Ruijin Hospital, Shanghai Jiao Tong University School of Medicine, Shanghai 200025 China; 2 Department of Rheumatology, Renji Hospital, Shanghai Jiao Tong University School of Medicine, Shanghai 200001 China; 3 Department of Radiology, Ruijin Hospital, Shanghai Jiao Tong University School of Medicine, Shanghai 200025 China; * Equal contribution

**Keywords:** hepatocellular carcinoma, Foxp3, DNMT1, methylation

## Abstract

Capability of tumor cells to impede immune response are largely associated with their interaction and regulation of CD4+CD25+ forkhead box transcription factor (Foxp)3+ regulatory T (Treg) cells, which suppress cytotoxic T cell-mediated immunity in the tumor microenvironment. Foxp3 level is critical for development and phenotypic maintenance of Treg, and is regulated by transcriptional control and epigenetic modification. Here, we showed that higher percentage of intratumoral Treg cells was positively correlated with lower Foxp3 promoter methylation in hepatocellular carcinoma (HCC), and both of them were associated with higher tumor grade, larger tumors, and poor prognosis of the patients. We used an adeno-associated virus (AAV) carrying either DNA (cytosine-5)-methyltransferase 1 (DNMT1) or shDNMT1 under a CD4 promoter (AAV-pCD4-DNMT1, AAV-pCD4-shDNMT1) to successfully target T-cells and alter the levels of DNMT1. Intratumoral injection of AAV- pCD4-DNMT1 significantly reduced tumor growth in mice, while intratumoral injection of AAV- pCD4-DNMT1 significantly induced tumor growth, compared to injection of control AAV. Finally, the effects of altering DNMT1 levels in T-cells seemed to affect tumor growth through alteration of methylation status of Foxp3 on promoter and CpG regions. Together, these data suggest that epigenetic control of Foxp3 in intratumoral T cells regulates growth of HCC.

## INTRODUCTION

Accumulating data suggest that tumor cells are capable of developing a variety of strategies to impede immune responses, which allows the tumor cells to grow and invade with exemption from the attacks from immune cells in the body [1]. Many of these strategies that tumor cells use to escape killing are associated with their interaction and regulation of regulatory T (Treg) cells [2]. Treg cells are primarily defined as a unique CD4+ T-cell lineage that plays a pivotal role in the maintenance of immunological tolerance [3]. Treg cells highly express CD25 [3]. Moreover, Treg cells are characterized by the expression of the forkhead box transcription factor Foxp3, which is not only a lineage-specifying factor, but also exerts specific functionality associated with immunosuppression in this T-cell subpopulation [3].

Treg cells are either recruited by solid tumor cells to migrate into the tumor niche, or are induced to differentiate from intratumoral undifferentiated T-cells [4–6]. Treg cells reversibly suppress cytotoxic T cell-mediated immunity in the tumor microenvironment, resulting in outgrowth and increased invasiveness of the tumor [7].

Stably transcriptionally regulation of Foxp3 gene expression is required for keeping a Treg cell lineage. Moreover, epigenetic modifications of Foxp3 at promoter, enhancer and a CpG region, primarily through methylation and demethylation, appears to be a critical regulatory mechanism for the expression of Foxp3 [8]. Hepatocellular carcinoma (HCC) is one of 5 tumors with the highest morbidity and one of the 3 tumors with the highest mortality [9]. The epigenetic regulation of Foxp3 has been associated with the tumorigenesis and prognosis of a number of cancers, including gastric cancer, colorectal carcinoma, endometrial cancer and lung cancer [1, 10, 11], but not studied in HCC so far.

In the current study, we showed that in HCCs, higher percentage of intratumoral Treg cells was positively correlated with lower Foxp3 promoter methylation, and both of them were associated with higher tumor grade, larger tumors, and poor prognosis of the patients. DNA (cytosine-5)-methyltransferase 1 (DNMT1) is a major methylation inducer [12]. We used an adeno-associated virus (AAV) carrying either DNMT1 or shDNMT1 under a CD4 promoter (AAV-pCD4-DNMT1, AAV-pCD4-shDNMT1) to successfully target T-cells and alter the levels of DNMT1. Intratumoral injection of AAV- pCD4-DNMT1 significantly reduced tumor growth in mice, while intratumoral injection of AAV- pCD4-DNMT1 significantly induced tumor growth, compared to injection of control AAV. Finally, the effects of altering DNMT1 levels in T-cells seemed to affect tumor growth through alteration of methylation status of Foxp3 on promoter and CpG regions.

## RESULTS

### More intratumoral Treg cells are detected in high-grade, large HCCs and correlate with poor prognosis

**Figure 1:**
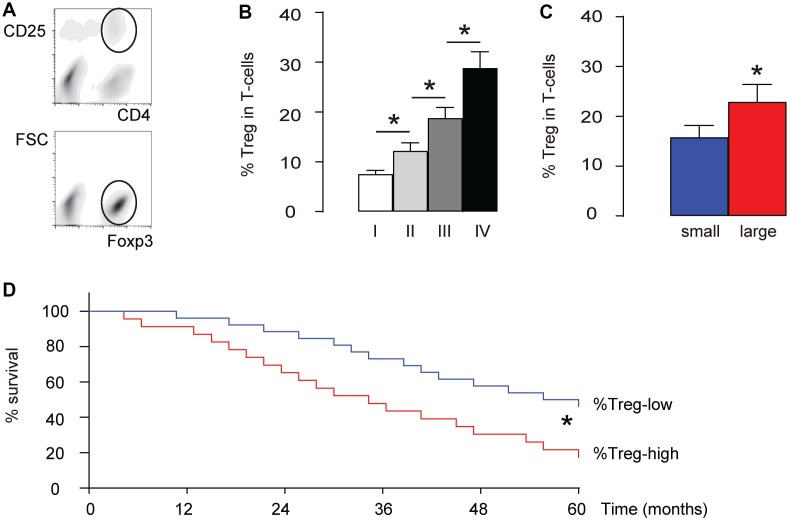
**More intratumoral Treg cells are detected in high-grade, large HCCs and correlate with poor prognosis.** We examined intratumoral Treg cells (CD4+CD25+Foxp3+) in total T-cells (CD4+) in 40 HCC specimens. (**A**) Illustration of FAC soring of CD4+CD25+Foxp3+ cells. First, CD4+CD25+ cells were isolated (circled gating in the upper panel), and this population was further purified for Foxp3+ cells (circled gating in the lower panel). (**B**) Percentage of intratumoral Treg cells in specimens with different tumor grades. (**C**) The median size of these 40 dissected tumors was used as a cutting point to get 20 small-size HCCs and 20 large-size HCCs. The percentage of intratumoral Treg cells in the large-size HCCs and small-size HCCs was compared. (**D**) The median level of %Treg was used to separate %Treg-high (n=20) from %Treg-low patients (n=20) to compare their overall five-year survival. *p<0.05. N=40.

We examined intratumoral Treg cells (CD4+CD25+Foxp3+) in total T-cells (CD4+) in 40 HCC specimens (Table 1, Figure 1A). We found that the percentage of intratumoral Treg cells significantly increased with the tumor grade (IV>III>II>I, p<0.05, Figure 1B). The median size of these 40 dissected tumors was used as a cutting point to get 20 small-size HCCs and 20 large-size HCCs. We found that the percentage of intratumoral Treg cells in the large-size HCCs was significantly higher than those from the small-size HCCs (p<0.05, Figure 1C). Next, we used the median level of %Treg to separate %Treg-high (n=20) from %Treg-low patients (n=20) and examined their overall five-year survival. We found that the overall survival of %Treg-low patients was significantly better (p<0.05, Figure 1D). Thus, more intratumoral Treg cells are detected in high-grade, or large HCCs and correlate with poor prognosis.

**Table 1:** Clinical-pathological characteristics (total)

	**Patients (n; %)**	**P value**
HCC tissue/tumor-adjacent normal tissue (NT)	40 (100%)/40 (100%)	
Age (<60/≥60 years old)	22 (55%)/18 (45%)	0.52
Gender (male/female)	28 (70%)/12 (30%)	
Tumor site (liver)	40 (100%)	
Tumor size (small/big)	20 (50%)/20 (50%)	0.011
Tumor stage (I/II/III/IV)	8 (20%)/16 (40%)/10 (25%)/6 (15%)	0.02
Lymph node metastasis (no/yes)	12 (30%)/28 (70%)	0.01
Distal metastasis at diagnosis (no/yes)	24 (60%)/16 (40%)	0.02

### Lower Foxp3 promoter methylation is detected in intratumoral T-cells from high-grade, large HCCs and correlate with %Treg in T-cells

**Figure 2:**
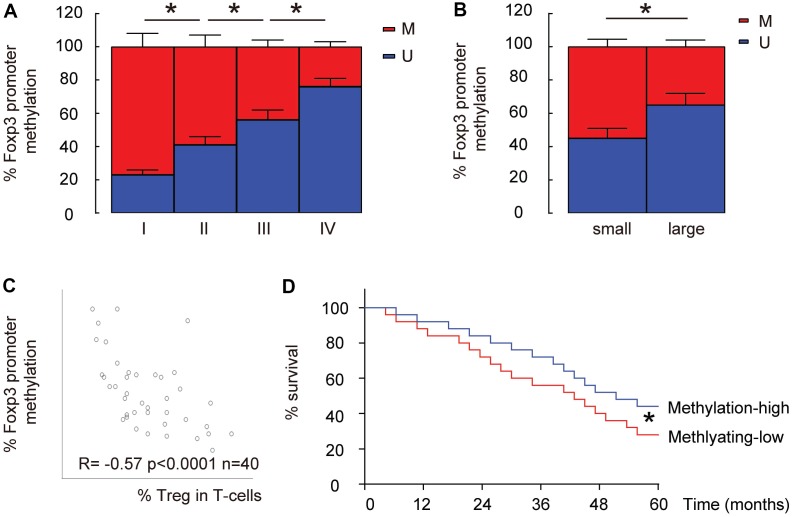
**Lower Foxp3 promoter methylation is detected in intratumoral T-cells from high-grade, large HCCs and correlate with %Treg in T-cells.** (**A**) The relative methylation levels of Foxp3 promoter in intratumoral T-cells in HCC specimens with different tumor grades by MS-PCR. (**B**) The relative methylation levels of Foxp3 promoter in intratumoral T-cells in small versus large HCCs by MS-PCR. (**C**) Correlation between the methylation levels of Foxp3 promoter and the percentage of Treg cells in total intratumoral T-cells. (**D**) The median level of methylation level of Foxp3 promoter was used to separate methylaition-high (n=20) from methylation-low patients (n=20) to compare their overall five-year survival.*p<0.05. N=40.

Since the epigenetic control (mainly through methylation) of Foxp3 promoter determines Treg differentiation and stability, we examined the levels of promoter methylation of Foxp3 in intratumoral T-cells in these 40 specimens. We found that the methylation levels of Foxp3 promoter in intratumoral T-cells significantly decreased with the tumor grade (IV<III<II<I, p<0.05, Figure 2A). The median size of these 40 dissected tumors was used as a cutting point to get 20 small-size HCCs and 20 large-size HCCs. The methylation levels of Foxp3 promoter in intratumoral T-cells from the large-size HCCs were significantly lower than those from the small-size HCCs (p<0.05, Figure 2B). Moreover, a significant inverse correlation was detected between the methylation levels of Foxp3 promoter and the percentage of Treg cells in total intratumoral T-cells (p<0.0001, R=-0.57, Figure 2C). Thus, Lower Foxp3 promoter methylation is detected in intratumoral T-cells from high-grade, large HCCs and correlate with %Treg in T-cells. Finally, we used the median level of promoter methylation of Foxp3 in intratumoral T-cells to separate methylation-high (n=20) from methylation-low patients (n=20) and examined their overall five-year survival. We found that the overall survival of methylation-low patients was significantly better (p<0.05, Figure 2D).

### Specific target and alteration of DNMT1 levels in T-cells

**Figure 3:**
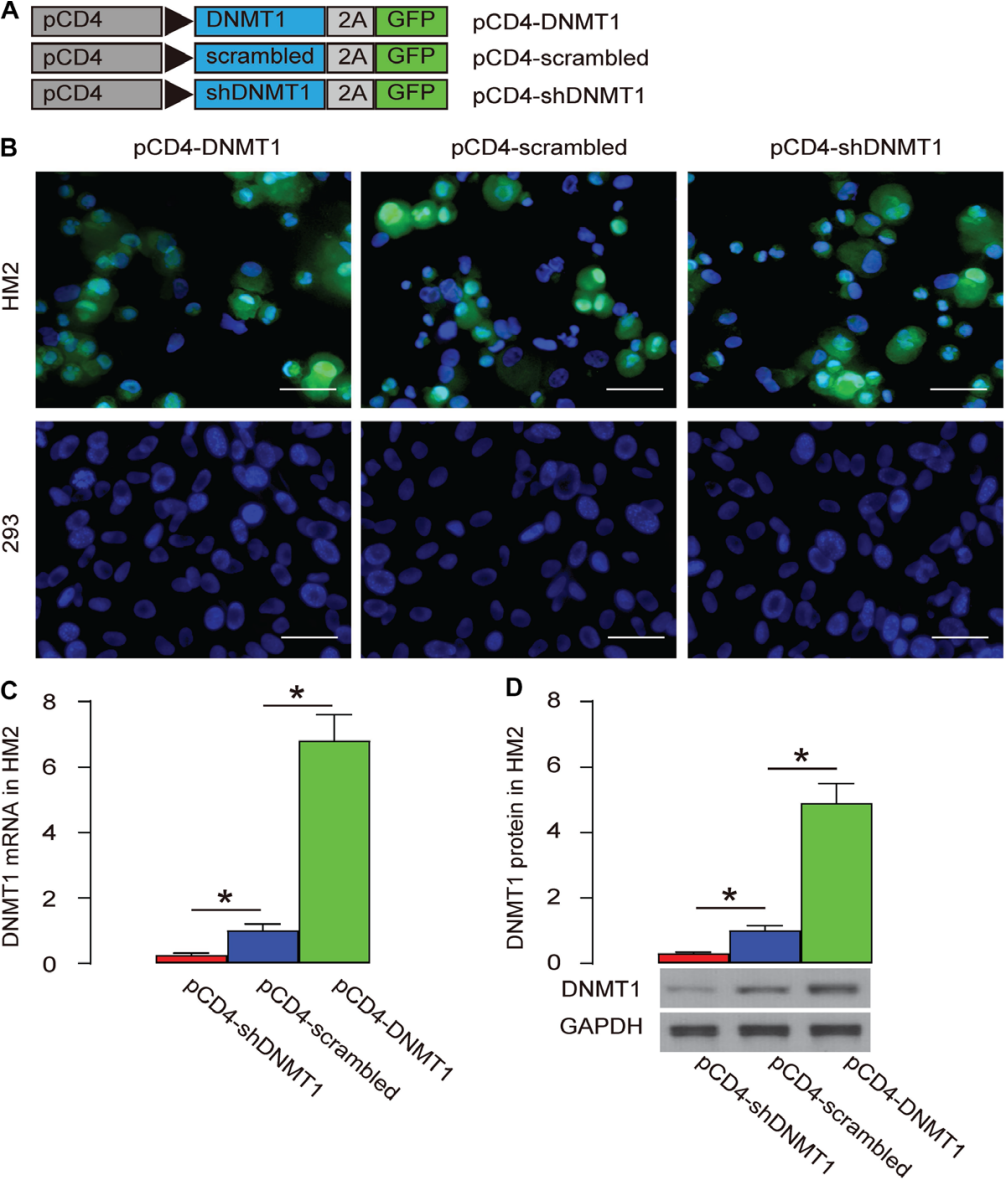
**Specific target and alteration of DNMT1 levels in T-cells.** (**A**) We used an AAV carrying either DNMT1, or scrambled (as a control) or shDNMT1 under a CD4 promoter (AAV-pCD4-DNMT1, AAV-pCD4-scrambled, AAV-pCD4-shDNMT1) to successfully target T-cells and alter the levels of DNMT1. (**B**) Direct fluorescence for GFP in transduced cells. (**C**, **D**) RT-qPCR (**C**) and Western blot (**D**) for DNMT1. *p<0.05. N=5. Scale bars are 20 μm.

DNA (cytosine-5)-methyltransferase 1 (DNMT1) is a major methylation inducer. We used an adeno-associated virus (AAV) carrying either DNMT1, or scrambled (as a control) or shDNMT1 under a CD4 promoter (AAV-pCD4-DNMT1, AAV-pCD4-scrambled, AAV-pCD4-shDNMT1) to successfully target T-cells and alter the levels of DNMT1 (Figure 3A). These AAVs were used to infect a human T-cell line, HM2 and a human non-T-cell, HEK293. We found that HM2 cells were transduced, while HEK293 cells were not, based on expression of GFP reporter (Figure 3B.). The changes in DNMT1 levels were assessed by RT-qPCR (Figure 3C), and by Western blot (Figure 3D), confirming the effects of DNMT1 overexpression or depletion.

### Epigenetic alteration in intratumoral T-cells affects tumor growth

**Figure 4:**
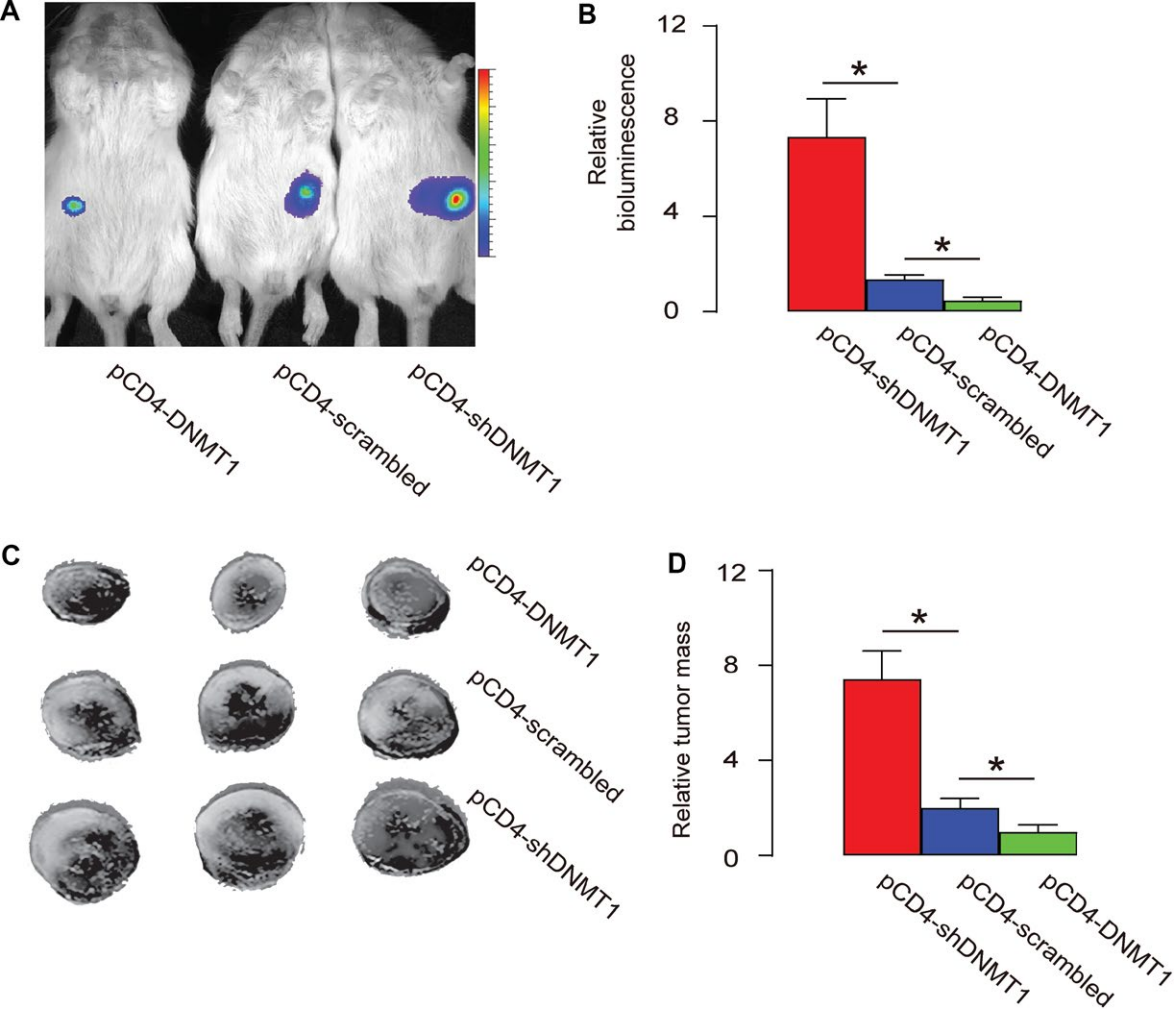
**Epigenetic alteration in intratumoral T-cells affects tumor growth**. A mouse HCC cell line Hepa1-6 that expresses luciferase reporter was subcutaneously implantated into C57/BL6 mice to generate detectable tumor. From the second week after transplantation, intratumoral injection with pAAV-pCD4-DNMT1, or pAAV-pCD4-scrambled, or pAAV-pCD4-shDNMT1 was done every week till 8 weeks, when the mice were sacrificed. (**A**–**B**) The bioluminescence analysis at sacrifice, shown by representative images (**A**), and by quantification (**B**). (**C**–**D**) Measurement of dissected tumor mass, shown by representative images (**C**), and by quantification (**D**). *p<0.05. N=3.

A mouse HCC cell line Hepa1-6 that expresses luciferase reporter was subcutaneously implantated into C57/BL6 mice to generate detectable tumor. From the second week after transplantation, intratumoral injection with AAV-pCD4-DNMT1, or AAV-pCD4-scrambled, or AAV-pCD4-shDNMT1 was done every week till 8 weeks, when the mice were sacrificed. The bioluminescence analysis showed that intratumoral injection with AAV-pCD4-DNMT1 significantly educed tumor size, while intratumoral injection with AAV-pCD4-shDNMT1 significantly increased tumor size, by representative images (Figure 4A), and by quantification (Figure 4B). Measurement of dissected tumor weights showed that intratumoral injection with AAV-pCD4-DNMT1 significantly reduced tumor mass, while intratumoral injection with AAV-pCD4-shDNMT1 significantly increased tumor mass, by representative images (Figure 4C), and by quantification (Figure 4D). Together, these data suggest that epigenetic alteration in intratumoral T-cells affects tumor growth.

### Promoter and CpG regions are the major sites where methylation is regulated by DNMT1

**Figure 5:**
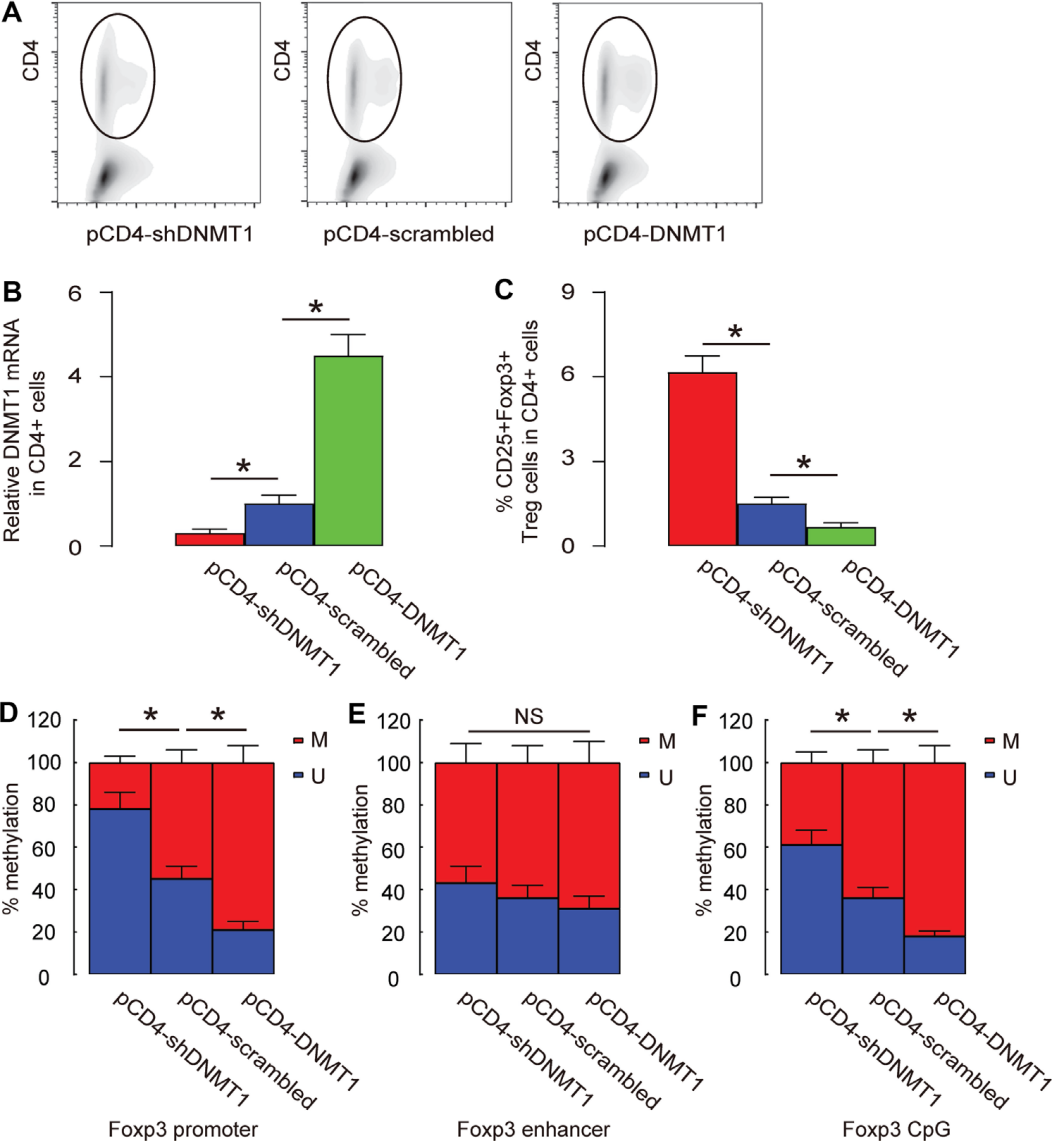
**Promoter and CpG regions are the major sites where methylation is regulated by DNMT1**. (**A**) Intratumoral T-cells were purified by CD4-based flow cytometry from the HCC-bared mice that had received intratumoral injection with AAV-pCD4-DNMT1, or AAV-pCD4-scrambled, or AAV-pCD4-shDNMT1. (**B**) RT-qPCR for DNMT1 in CD4+ cells from AAV-pCD4-DNMT1-, or AAV-pCD4-scrambled-, or AAV-pCD4-shDNMT1- injected tumor. (**C**) % CD4+CD25+Foxp3+ Treg cells in total CD4+ cells. (**D**–**F**) Methylation status of 3 known sites on Foxp3 (promoter (**D**), enhancer (**E**) and CpG (**F**) region) were assessed by MS-PCR. *p<0.05. NS: non-significant. N=3.

Foxp3 has 3 known sites that are susceptible for methylation, promoter, enhancer and CpG region. We analyzed the changes in methylation status at these sites in intratumoral T-cells by DNMT1 alterations. Intratumoral T-cells were purified by CD4-based flow cytometry (Figure 5A). First, we examined the DNMT1 levels in CD4+ T-cells and found that intratumoral injection with AAV-pCD4-DNMT1 significantly increased DNMT1 levels in intratumoral T-cells, while intratumoral injection with AAV-pCD4-shDNMT1 significantly decreased DNMT1 levels in intratumoral T-cells (Figure 5B). Moreover, CD4+CD25+Foxp3+ Treg cells were quantified in tumors and showed significant increase in AAV-pCD4-shDNMT1-injected tumor but significant decrease in AAV-pCD4-DNMT1-injected tumor (Figure 5C). We found that the methylation status of Foxp3 on promoter (Figure 5D) and CpG regions (Figure 5F), but not the enhancer region (Figure 5E), was significantly altered by pAAV-pCD4-DNMT1 or pAAV-pCD4-shDNMT1.

## DISCUSSION

The non-tumor cells in the tumor environment play a critical role in the tumor growth, metastasis and tumor immunology [13]. These cells include endothelial cells, mesenchymal cells, neural cells and immune cells comprised primarily of macrophages and T-cells. Recently, the role of Treg, a specific subpopulation with inhibitory effects on immune reaction, has been recognized [13]. Foxp3 is a signature and most important determinant for Treg, while its expression is controlled by both transcription and epigenetic modification, with the latter appears to be more important [1].

The epigenetic modifications, including methylation/demethylation, acetylation/deacetylation, sumoylation/desumoylation, etc, play a substantial role in tumorigenesis [8]. However, different modification on different cell types may determine the eventual effects on the outcome of carcinogenesis. For example, the effects of promoter methylation on oncogenes may have a suppressive effect on tumor growth, while the effects of promoter methylation on tumor suppressive genes may have a promoting effect on tumor growth [8]. Thus, in the current study, we used a T-cell-specific CD4 promoter to drive the methylation controlling gene, DNMT1, or its shRNA [12]. This approach allowed us to specifically target T-cells, and studied the specific epigenetic modification on intratumoral T-cells through in situ injection, and left tumor cells unaffected. Although some intra-tumoral immune cells other than T-cells may also express CD4, the levels of CD4 in these cells are very low, compared to T-cells. Moreover, these populations are minor. Therefore, we think that the effects of presence of these CD4+ non-T cells on our results should be minimal.

Our findings suggest that increased methylation on T-cells was beneficial. This outcome should be largely resulting from the effects on Foxp3 promoter. The Foxp3 has 3 sites that are important for expression control and are regulated by methylation. The first site is the promoter, which is a classic TATA and CAAT- box-containing region located 6.5 kb upstream of exon one [8]. Demethylation of this promoter site opens binding sites for activator protein 1 (AP-1), signal transducer and activator of transcription 5 (Stat5), nuclear factor of activated T cells (NFAT) and TGFβ-inducible early gene 1 (TIEG1) [8]. The second site is at the enhancer, and works as a TGFβ sensor, since it is a TGFβ-sensitive element with binding sites for NFAT and mothers against decapentaplegic homologues (SMADs) [8]. We did not find that the methylation of this region by DNMT1, probably due to that this region is also regulated by acetylation [14]. The third site is a Treg-cell-specific demethylated region characterized with highly conserved CpG-rich nuclear acids [8]. This region was already known to be fully demethylated in Treg cells and fully methylated in non-Treg T-cells [8]. Here, we found that the methylation status of this region was controlled by DNMT1. The status of the methylation in this region and the first promoter region appeared to be functionally associated with Treg cell differentiation and tumor growth. Previous studies have demonstrated that the third region may be essential for Treg cell stability [15], suggesting that the stability of intratumoral Treg cells may be regulatable through epigenetic modifications.

Here, we used a mouse HCC cell line to generate implanted tumor model in immune-competent mice rather than using immune-deficient nude mice, since we need regular immunity to test the epigenetic modification of T-cells on the outcome of the tumor growth. The use of CD4 promoter and intratumoral injection of AAVs restricted the interference on intratumoral T-cells. Future studies may address the different roles of different Foxp3 methylation regions on Treg biology and their functionality on tumor growth.

## MATERIALS AND METHODS

### Animal and experimental protocols

All the experimental methods including animal experiments have been approved by the research committee and the Institutional Animal Care and Use Committee at the Shanghai Jiao Tong University School of Medicine.

### Human samples

The research work on human samples was done following these guidelines and in accordance with the instruction from institutional Ethics Committee. Surgically removed specimens of HCC were obtained from 40 patients who underwent surgery between 2011 and 2013 at Ruijin Hospital (Table 1). Fresh tissue was digested with 0.25% Trypsin (Sigma-Aldrich, San Jose, CA, USA) for 45 minutes, followed by T-cell (CD4+) and Treg cell (CD4+CD25+Foxp3+) isolation by flow cytometry. Total CD4+ cell population was used for promoter methylation analysis.

### Cell culture and treatment

A human T-cell line HM2, a human cell line HEK293, and a mouse HCC cell line Hepa1-6 were all purchased from ATCC (American Type Culture Collection, Manassas, VA, USA). These cells were cultured in Dulbecco's Modified Eagle's Medium supplemented with 10 % Fetal Bovine Serum (FBS, Invitrogen, Carlsbad, CA, USA) in a 37 °C incubator with an atmosphere of 5 % CO_2_.

### Plasmids, AAVs, and cell transfection

DNMT1-coding sequence was amplified from cDNA from human bone marrow. CD4 promoter was cloned from human genomic DNA. Short-hairpin small interfering RNA for DNMT1 (shDNMT1) used the targeting sequence of “5′-GCCCAATGAGACTGACATCAA-3′”. A scrambled sequence (5′-CACCGAGCCCACCACAGCTCAAG-3′) was used as a control. The plasmids for generating vectors were prepared from a pAAV-CAG-GFP plasmid (Clontech, Mountain View, CA, USA). By molecular cloning assay, CAG promoter was replaced with CD4 promoter. DNMT1, or scrambled, or shDNMT1 with a 2A sequence (allows equally expression of 2 genes by one promoter) was inserted immediately before GFP reporter, to generate pAAV-pCD4-DNMT1, or pAAV-pCD4-scrambled, or pAAV-pCD4-shDNMT1. Transfection of HM2 or HEK293 cells was performed using Lipofectamine 3000 (Invitrogen), as instructed. AAVs were prepared with a packaging kit (Clontech). Hepa1-6 cells were transduced with an AAV-pCMV-luciferase (Clontech) to allow in vivo detection in the live animals.

### Tumor graft model in mice

The male C57/BL6 mice at 12-week-old (SLAC Laboratory Animal Co. Ltd, Shanghai, China) were used for subcutaneous implantation of 10^6^ AAV-pCMV-luciferase-transduced mouse Hepa1-6 cells at their flanks (n=3 for each group). From the second week after transplantation, intratumoral injection with 10^11^ viral particles pAAV-pCD4-DNMT1, or pAAV-pCD4-scrambled, or pAAV-pCD4-shDNMT1 was done every week till 8 weeks, when the mice were sacrificed and the bioluminescence and the size of the dissected tumor weights were measured. The bioluminescence detection was performed and the images were recorded with IVIS imaging system (Xenogen Corp., Alameda, CA, USA), with an acquisition time of 60-second and binning of 10.

### Flow cytometry

Tumor was digested with 0.25% Trypsin (Sigma-Aldrich) for 45 minutes, followed by flow cytometry analysis and cell sorting. The cell preparations were labeled with PE-conjugated anti-CD4 antibody, APC-conjugated anti-CD25 antibody and Pacific blue-conjugated anti-CD4 antibody (Becton-Dickinson Biosciences, San Jose, CA, USA), GFP was detected by direct fluorescence. Flow cytometry was performed using a FACSAria (Becton-Dickinson Biosciences) flow cytometer, and the data were analyzed and presented using Flowjo software version 10 (Flowjo LLC, Ashland, OR, USA).

### Quantitative reverse transcription polymerase chain reaction (RT-qPCR) and methylation-specific PCR (MS-PCR)

TaqMan quantitative PCR (RT-qPCR, two step) was applied to examine the relative levels of the genes, using GAPDH as an internal control. The total RNA was isolated using TRIzol® reagent (Invitrogen), according to the manufacturer’s instructions. RNA was then reverse transcribed into cDNA using the High Capacity cDNA Reverse Transcription kit (Applied Biosystems, Foster City, CA, USA). TaqMan Gene Expression Assays were done using pre-designed primers on an ABI 7500 Fast Real-time PCR system (Applied Biosystems). Relative quantification of gene expression was performed using the 2-ΔΔCt method.

MS-PCR was performed to identify the methylation status of Foxp3. Bisulfite conversion of DNA was done with an EZ-96 DNA Methylation-Gold kit (Zymo Research, Irvine, CA, USA). Each 15 ml MS-PCR included 7.5 ml Qiagen Multiplex Master Mix (Qiagen, Hilden, Germany), 3 ml Q solution (Qiagen), 0.3 ml forward primers, 0.3 ml reverse primers, 2.9 ml of RNAse-free water and 1 ml of bisulfite-converted template DNA. The primer sequences used in the MS-PCR were shown in Table 2. MS-PCR products were analyzed by agarose gel electrophoresis. Samples were defined as methylated or unmethylated depending upon the visual band amplified with methylated or unmethylated primers.

**Table 2:** Sequences of primers used for MS-qPCR for Foxp3

**Primers for detection of**	**Primer type**	**Sequence (5′ to 3′)**
Methylated FOXP3-promoter	Sense	CTCTTCTCTTCCTCCGTAATATCG
	Antisense	GTTATTGACGTTATGGCGGTC
Demethylated FOXP3-promoter	Sense	CCCTCTTCTCTTCCTCCATAATATCA
	Antisense	TTTTGTTATTGATGTTATGGTGGTT
Methylated FOXP3-enhancer	Sense	GTAAAGGGTAGTTGGAAGGTAAAGC
	Antisense	AGAGGTTTAAAAAGTGGGAGATTTC
Demethylated FOXP3-enhancer	Sense	GTACGAACCTCACACGACGA
	Antisense	TAAAGGGTAGTTGGAAGGTAAAGTG
Methylated FOXP3-CpG	Sense	TACACATACAAACCTCACACAACAA
	Antisense	ATTAACTCGCTACAACCATTATCGT
Demethylated FOXP3-CpG	Sense	AGAGGTTTAAAAAGTGGGAGATTTT
	Antisense	TTAACTCACTACAACCATTATCATC

### Western blot

Cells were lysed in lysis buffer (Sigma-Aldrich) for 30 minutes. A BCA kit (Pierce Biotechnology, Rockford, IL, USA) was used to determine protein concentrations. After gel electrophoresis, membrane transfer and blocked with 5% non-fat milk, the blots were probed with antibodies against DNMT1 (Abcam, Beijing, China) and GAPDH (Cell signaling, Shanghai, China) overnight. The blots were then washed and incubated with horseradish peroxidase-conjugated secondary antibodies (Santa Cruz Biotechnology, Inc., Nanjing, China) for 1 hour at room temperature, followed by development of the signal by an ECL detection reagent (Pierce Biotechnology) and quantification with densitometry using Image J software (Bethesda, MA, USA). Blotting images were representative from 5 repeats.

### Statistical analysis

GraphPad Prism 7 (GraphPad, Chicago, IL, USA) software was used in the current study. The methods include one-way and two-way ANOVA with a Bonferroni correction, followed by Fisher’s Exact Test for comparison between two groups. All values are depicted as mean ± standard deviation and are considered significant if p < 0.05. Bivariate correlations were calculated by Spearman's rank correlation coefficients. Kaplan-Meier curve was applied to record the overall survival of the patients included in this study.
